# Analysis of blood culture in a rat model of cecal ligation and puncture induced sepsis

**DOI:** 10.1186/s40635-020-00310-6

**Published:** 2020-06-05

**Authors:** Prabakar Vaittinada Ayar, Hervé Jacquier, Benjamin Deniau, Feriel Azibani, Alexandre Mebazaa, Alice Blet

**Affiliations:** 1 Emergency Department, Beaujon Hospital, AP-HP, 100, Boulevard du Général Leclerc, 92300 Clichy, France; 2 INSERM UMR-S942 MASCOTT, Paris, France; 3 University of Paris, Paris, France; 4 Laboratory of Microbiology, Department of Infectious Agents, Lariboisière Hospital, AP-HP, Paris, France; 5 INSERM, IAME, UMR 1137, Paris, France; 6 GH St-Louis-Lariboisière, Department of Anesthesiology, Critical Care and Burn Unit, St-Louis Hospital, AP-HP, Paris, France

To the Editor,

Sepsis shows a high incidence and is associated with a high mortality [1, 2], and experimental studies are useful for a better understanding of sepsis and for identifying new therapies [3].

The cecal ligation and puncture (CLP) model is considered as the gold standard experiment for studying sepsis in animals. Recent guidelines recommend resuscitating animals after performing CLP including administration of fluid and chosen antimicrobials based on known pathogens. However, microbial identification is not a common practice in pre-clinical models of sepsis.

The aims of this original study were:

- To assess microbial situation in CLP-induced sepsis in the recent literature
- To document microbiology of blood cultures in rat CLP-induced sepsis performed in our lab.

## Keyword-based review

The review was performed on PubMed using “CLP” and “rat” keywords for English-written papers in 2018 and 2019 and also for “mouse” and “CLP.” Bacteriological documentation and antimicrobial therapy were collected.

## CLP model in rats

Sixteen Wistar male rats, from 9 to 12 weeks of age weighing 350 to 450 g were obtained from Janvier (St. Berthevin, France). CLP model was performed as previously described [4, 5]. Septic shock was reached 16 h after induction by CLP, and blood samples were collected by jugular withdrawn. Culture and bacteriological analysis were done as previously described [6, 7].

## Keyword-based review revealed a few administrations of antibiotics in CLP models

Our keyword-based review performed on PubMed resulted in 176 publications between 2018 and 2019 for rats. Among them, 22 (12.5%) were excluded for a different meaning of CLP acronym. In only 15% (23/154), antimicrobial therapy has been used, mostly (57%) the third generation cephalosporin (ceftriaxone) (Table S1).

For mice, 59 (14.6%) of 405 studies were excluded. In 18% (62/346), antibiotics have been administered, mostly (47/62, 76%) the carbapenem (imipenem/ertapenem) (Table S2).

However, none of the studies performed microbiological documentation before treatment.

## Blood culture analysis 16 h after sepsis induction

In 16 CLP rats, *Escherichia coli* 88% (14/16), *Enterococcus faecalis* 81% (13/16), and *Enterobacter cloacae* 75% (12/16) were the main pathogens found in blood cultures (Table 1). All bacteria exhibit a wild-type phenotype for antimicrobial agent susceptibility.

**Table 1:**
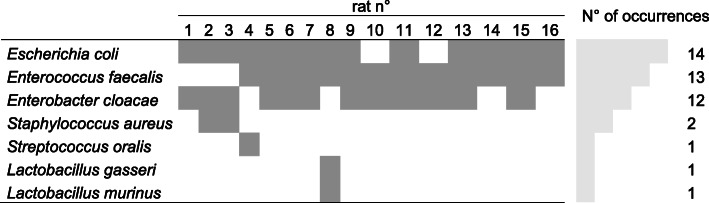
Blood culture analysis of 16 rats 16 h after CLP

Our literature review about CLP-induced sepsis showed that antibiotherapy and bacteriological documentation was not reported in experimental models.

Blood cultures in our CLP model frequently identified 3 bacteria, in accordance with common polymicrobial infections in stercoral peritonitis in humans. Further, similar microbial profile (*Enterobacteriaceae* and *Enterococci)* was also found between our CLP model and human peritonitis [8].

By analyzing antimicrobial susceptibility testing, all bacteria exhibit a wild-type phenotype. Carbapenems definitely proved to be the most congruent antibiotics in our model. However, antimicrobial narrow spectrum therapy, including cotrimoxazole, seemed appropriate (Table 2).

**Table 2:** In vitro susceptibility (minimal inhibitory concentration (μg/mL)) of the organisms identified in the blood culture in our CLP rats (*n* = 16) to antimicrobial drugs

	*E. coli*	*E. cloacae*	*E. faecalis*
Imipenem	0.38	0.38	1
Tazocillin	2	2	2
Cotrimoxazole	0.6	0.125	0.016
Levofloxacin	0.06	0.06	1

To conclude, our literature search shows that antimicrobial therapy is not daily used in the treatment of CLP-induced sepsis, and when used, no bacterial identification is performed. Our data indicates that blood culture is readily available and may give a correct indication on which antimicrobial therapy to use in CLP-induced sepsis.

## Supplementary information

**Additional file 1: Figure S1.** Peritoneal fluid culture analysis of 16 rats 16 hours after CLP

**Additional file 2: Table S1.** Probabilistic antimicrobial therapy used in rat CLP models in the literature published in 2018 and 2019

**Additional file 3: Table S2.** Probabilistic antimicrobial therapy used in mouse CLP models in the literature published in 2018 and 2019

## Data Availability

The datasets used and/or analyzed during the current study are available from the corresponding author on reasonable request.

## Abbreviation

CLP: Cecal ligation and puncture

## References

[1] Fleischmann C, Scherag A, Adhikari NKJ (2016). Assessment of global incidence and mortality of hospital-treated sepsis. Current Estimates and Limitations. Am J Respir Crit Care Med.

[2] Rudd KE, Johnson SC, Agesa KM, et al (2020) Global, regional, and national sepsis incidence and mortality, 1990–2017: analysis for the Global Burden of Disease Study. Lancet 395:200–211. 10.1016/S0140-6736(19)32989-7 10.1016/S0140-6736(19)32989-7 PMC6970225 31954465

[3] Osuchowski MF, Ayala A, Bahrami S (2018). Minimum quality threshold in pre-clinical sepsis studies (MQTiPSS): an international expert consensus initiative for improvement of animal modeling in sepsis. Intensive Care Med Exp.

[4] Rittirsch D, Huber-Lang MS, Flierl MA, Ward PA (2009). Immunodesign of experimental sepsis by cecal ligation and puncture. Nat Protoc.

[5] Blet A, Deniau B, Geven C (2019). Adrecizumab, a non-neutralizing anti-adrenomedullin antibody, improves haemodynamics and attenuates myocardial oxidative stress in septic rats. Intensive Care Med Exp.

[6] Amarsy-Guerle R, Mougari F, Jacquier H (2015). High medical impact of implementing the new polymeric bead-based BacT/ALERT® FAPlus and FNPlus blood culture bottles in standard care. Eur J Clin Microbiol Infect Dis.

[7] EUCAST The European Committee on Antimicrobial Susceptibility Testing. Breakpoint tables for interpretation of MICs and zone diameters, version 9.0, 2019. http://www.eucast.org/fileadmin/src/media/PDFs/EUCAST_files/Breakpoint_tables/v_9.0_Breakpoint_Tables.pdf. Accessed 27 Nov 2019

[8] Montravers P, Lepape A, Dubreuil L (2009). Clinical and microbiological profiles of community-acquired and nosocomial intra-abdominal infections: results of the French prospective, observational EBIIA study. J Antimicrob Chemother.

