# Supplementary figures and images for: Analysis of blood culture in a rat model of cecal ligation and puncture induced sepsis
Source: Intensive Care Med Exp. 2020 Jun 5;8:18. doi: 10.1186/s40635-020-00310-6 (PMC7275103; doi:10.1186/s40635-020-00310-6)

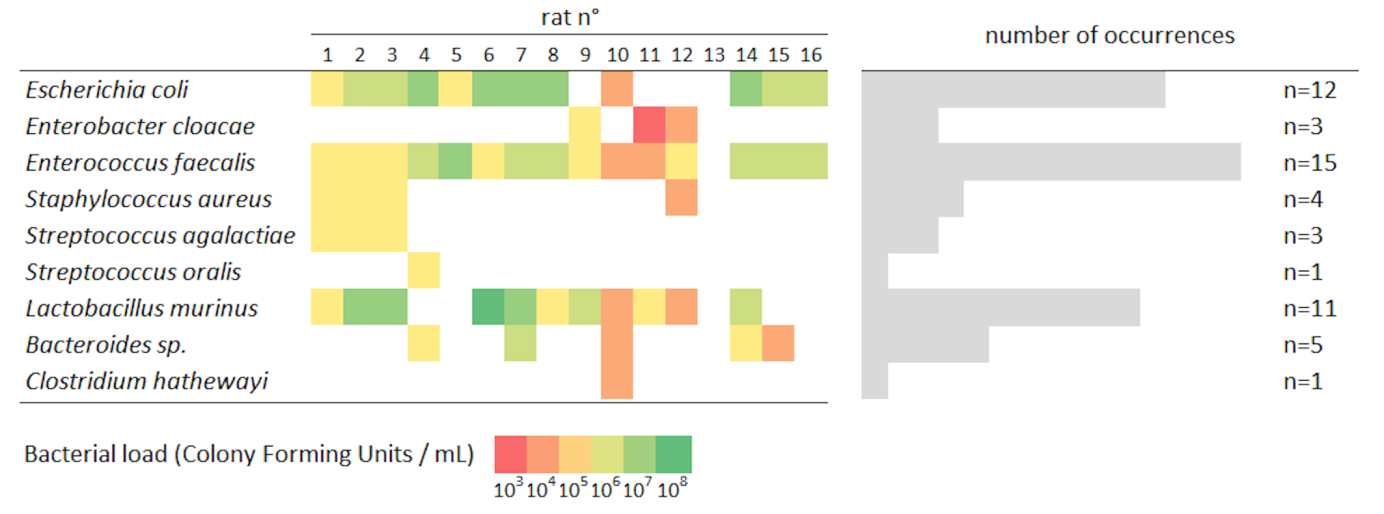


Figure S1. Peritoneal fluid culture analysis of 16 rats 16 hours after CLP

Supplement: Supplementary file 1 — Additional file 1: Figure S1. Peritoneal fluid culture analysis of 16 rats 16 hours after CLP [file 40635_2020_310_MOESM1_ESM.docx]
