# Supplementary material for: Analysis of blood culture in a rat model of cecal ligation and puncture induced sepsis
Source: Intensive Care Med Exp. 2020 Jun 5;8:18. doi: 10.1186/s40635-020-00310-6 (PMC7275103; doi:10.1186/s40635-020-00310-6)
Supplement: Supplementary file 2 — Additional file 2: Table S1. Probabilistic antimicrobial therapy used in rat CLP models in the literature published in 2018 and 2019 [file 40635_2020_310_MOESM2_ESM.docx]

Suppl Table1. Probabilistic antimicrobial therapy used in rat CLP models in the

literature published in 2018 and 2019

| Author  Year | Treatment, dose | Route of administration | Duration | Objective |
| --- | --- | --- | --- | --- |
| Assis MS ^1^  2018 | gentamycine 3 mg/kg + clindamycine 25 mg/kg | IP | 72 hours (dose per day not specified) | Effects of caffeine on behavioural and cognitive deficits in rats |
| Bedirli N ^2^  2018 | ceftriaxone 30 mg/kg + clindamycine 25 mg/kg | IP | every 6 hours for 72 h | To compare the effects of sevoflurane and isoflurane on systemic inflammation |
| Choi M ^3^  2018 | moxifloxacine 3 mg/kg | Non specified | Single dose after surgery | Effect of hypobaria and hyperoxia during sepsis on survival and energy metabolism |
| Danielski LG ^4^  2018 | ceftriaxone 30 mg/kg | SC | immediately after and 12 hours after CLP | Effect of vit B6 on the Kynurenine pathway, acute neurochemical and neuroinflammatory parameters, and cognitive dysfunction |
| Della Giustina A ^5^  2018 | ceftriaxone 30 mg/kg | SC | immediately after and 12 hours after CLP | Determine the effect of a fish oil(FO)-55–enriched lipid emulsion as an important anti-inflammatory compound on brain dysfunction |
| Gasparotto J ^6^  2018 | ceftriaxone 30 mg/kg et clindamycin 25 mg/kg | SC | every 6 hours for 72 hours | Determine role of receptor for advanced glycation end products (RAGE) in neuroinflammation, neurodegeneration-associated changes, and cognitive dysfunction arising after sepsis recovery |
| Giustina AD ^7^  2018 | ceftriaxone 30 mg/kg | SC | immediately after and 12 hours after CLP | Evaluate the effect of DMF (dimethyl fumarate) in different organs of rats submitted to an animal model of sepsis |
| Wang P ^8^  2018 | ceftriaxone 30 mg/kg | SC | every 6 hours for 72 hours | Investigate mechanisms and molecular pathways of neuroprotective effects of Omi/HtrA2 inhibition |
| Wang Y ^9^  2018 | imipenem/cilastatin 14 mg/kg | IP | Single dose at 6 hours after surgery | Determine whether resveratrol alleviates pediatric AKI and investigated the potential mechanism |
| Yang J ^10^  2018 | imipenem 120mg/kg | IP | Single dose | Effect of combination treatment of the sepsis bundle with cyclophosphamide would improve the function of the intestinal mucosa and enhance survival in rats with induced sepsis |
| Zarbato GF ^11^  2018 | ceftriaxone 30 mg/kg | SC | immediately after and 12 hours after CLP | Dimethyl Fumarate Limits Neuroinflammation and Oxidative Stress and Improves Cognitive Impairment after Polymicrobial Sepsis |
| Capcha JMC ^12^  2019 | imipenem/cilastatin 14 mg/kg | SC | Single dose at 6 hours after surgery | Evaluate the effects of theWharton’s jelly-derived mesenchymal stem cells as a treatment for cardiopulmonary injury, as well as on neuroimmunomodulation |
| Chen J ^13^  2019 | imipenem/cilastatin 25 mg/kg | IP | one time immediately after surgery | Investigate the role and possible mechanisms of exogenous melatonin to improve liver injury and glucose metabolism disturbances in the context of sepsis. |
| Jiang Y ^14^  2019 | imipenem 25 mg/kg | SC | one time immediately after surgery | Analyze the metabolic changes of rats sepsis model established by cecal ligation and puncture (CLP) and evaluate therapeutic targets and mechanisms of Xuebijing on sepsis |
| Liverani E ^15^  2019 | imipenem/cilastatin 25 mg/kg | IP | 2 times per day until euthanasia after surgery during 4 days | Investigate whether Protein kinase C‐delta inhibition provided organ protection and improved survival without compromising pathogen clearance. |
| Manfredi A ^16^  2019 | ceftriaxone 30 mg/kg | SC | Every 6 hours during 3 days | Determine whether stimulation of mitochondrial dynamics improves mitochondrial function and long-term cognitive impairment in an experimental model of sepsis |
| Michels M ^17^  2019 | ceftriaxone 30 mg/kg + clindamycin 25 mg/kg | SC | Every 6 hours during 3 days | Characterize the kinetics of microglial phenotypes early and late after systemic inflammation in an animal model of severe sepsis, and the effects of minocycline on these phenotypes |
| Michels M ^18^  2019 | ceftriaxone 30 mg/kg | SC | Every 12 hours during 3 days | Determine the effects of microglial depletion and subsequent repopulation early during systemic and brain inflammatory response and its impact on late cognitive dysfunction in an animal model of severe sepsis |
| Milioli MVM ^19^  2019 | ceftriaxone 30 mg/kg | SC | Every 6 hours during 3 days | Evaluate behavioral and neurochemical parameters in adult (180-day-old) and young (60-day-old) rats subjected to sepsis |
| Petronilho F ^20^  2019 | ceftriaxone 30 mg/kg | SC | immediately after and 12 h after CLP | Investigate if the association of gold nanoparticles and N-acétylcystéine could prevent oxidative stress and mitochondrial and creatine kinase alterations in the brain of septic rats |
| Wu J ^21^  2019 | imipenem 25 mg/kg | IP | twice a day for 3 days. | Investigate the protective effects of neuregulin-1β (NRG-1β) on sepsis-induced diaphragm atrophy and the possible underlying mechanisms |
| Yang Y ^22^  2019 | ceftriaxone 30 mg/kg | SC | Every 6 hours during 3 days | Investigated whether the anti-apoptotic effect of IGF-1 is associated with cytochrome C and TNFR regulation |
| Yang YM ^23^  2019 | imipenem 14 mg/kg | SC | Single dose at 6 hours after surgery | investigate the regulatory effect of long non-coding RNA (lncRNA) NKILA on autophagy in sepsis-induced kidney injury |

IP: intraperitoneal

SC: subcutaneous

[1–23]

1. Assis MS, Soares AC, Sousa DN, et al (2018) Effects of Caffeine on Behavioural and Cognitive Deficits in Rats. Basic & Clinical Pharmacology & Toxicology 123:435–442. https://doi.org/10.1111/bcpt.13036

2. Bedirli N, Bagriacik EU, Yilmaz G, et al (2018) Sevoflurane exerts brain-protective effects against sepsis-associated encephalopathy and memory impairment through caspase 3/9 and Bax/Bcl signaling pathway in a rat model of sepsis. J Int Med Res 46:2828–2842. https://doi.org/10.1177/0300060518773265

3. Choi M, Tamrakar P, Schuck PF, et al (2018) Effect of hypobaria and hyperoxia during sepsis on survival and energy metabolism: Journal of Trauma and Acute Care Surgery 85:S68–S76. https://doi.org/10.1097/TA.0000000000001909

4. Danielski LG, Giustina AD, Goldim MP, et al (2018) Vitamin B6 Reduces Neurochemical and Long-Term Cognitive Alterations After Polymicrobial Sepsis: Involvement of the Kynurenine Pathway Modulation. Mol Neurobiol 55:5255–5268. https://doi.org/10.1007/s12035-017-0706-0

5. Della Giustina A, Goldim MP, Danielski LG, et al (2018) Fish oil-rich lipid emulsion modulates neuroinflammation and prevents long-term cognitive dysfunction after sepsis. Nutrition 110417. https://doi.org/10.1016/j.nut.2018.12.003

6. Gasparotto J, Girardi CS, Somensi N, et al (2018) Receptor for advanced glycation end products mediates sepsis-triggered amyloid-β accumulation, Tau phosphorylation, and cognitive impairment. J Biol Chem 293:226–244. https://doi.org/10.1074/jbc.M117.786756

7. Giustina AD, Bonfante S, Zarbato GF, et al (2018) Dimethyl Fumarate Modulates Oxidative Stress and Inflammation in Organs After Sepsis in Rats. Inflammation 41:315–327. https://doi.org/10.1007/s10753-017-0689-z

8. Wang P, Hu Y, Yao D, Li Y (2018) Omi/HtrA2 Regulates a Mitochondria-Dependent Apoptotic Pathway in a Murine Model of Septic Encephalopathy. Cell Physiol Biochem 49:2163–2173. https://doi.org/10.1159/000493819

9. Wang Y, Feng F, Liu M, et al (2018) Resveratrol ameliorates sepsis-induced acute kidney injury in a pediatric rat model via Nrf2 signaling pathway. Exp Ther Med 16:3233–3240. https://doi.org/10.3892/etm.2018.6533

10. Yang J, Zhang S, Wu J, et al (2018) Imipenem and normal saline with cyclophosphamide have positive effects on the intestinal barrier in rats with sepsis. Biomed Pap Med Fac Univ Palacky Olomouc Czech Repub 162:90–98. https://doi.org/10.5507/bp.2018.032

11. Zarbato GF, de Souza Goldim MP, Giustina AD, et al (2018) Dimethyl Fumarate Limits Neuroinflammation and Oxidative Stress and Improves Cognitive Impairment After Polymicrobial Sepsis. Neurotox Res 34:418–430. https://doi.org/10.1007/s12640-018-9900-8

12. Capcha JMC, Rodrigues CE, Moreira R de S, et al (2019) Wharton’s jelly-derived mesenchymal stem cells attenuate sepsis-induced organ injury partially via cholinergic anti-inflammatory pathway activation. Am J Physiol Regul Integr Comp Physiol 318:R135–R147. https://doi.org/10.1152/ajpregu.00098.2018

13. Chen J, Xia H, Zhang L, et al (2019) Protective effects of melatonin on sepsis-induced liver injury and dysregulation of gluconeogenesis in rats through activating SIRT1/STAT3 pathway. Biomed Pharmacother 117:109150. https://doi.org/10.1016/j.biopha.2019.109150

14. Jiang Y, Zou L, Liu S, et al (2019) GC/MS-based metabonomics approach reveals effects of Xuebijing injection in CLP induced septic rats. Biomed Pharmacother 117:109163. https://doi.org/10.1016/j.biopha.2019.109163

15. Liverani E, Tursi SA, Cornwell WD, et al (2019) Protein kinase C-delta inhibition is organ-protective, enhances pathogen clearance, and improves survival in sepsis. FASEB J 34:2497–2510. https://doi.org/10.1096/fj.201900897R

16. Manfredini A, Constantino L, Pinto MC, et al (2019) Mitochondrial dysfunction is associated with long-term cognitive impairment in an animal sepsis model. Clin Sci 133:1993–2004. https://doi.org/10.1042/CS20190351

17. Michels M, Abatti MR, Ávila P, et al (2019) Characterization and modulation of microglial phenotypes in an animal model of severe sepsis. J Cell Mol Med 24:88–97. https://doi.org/10.1111/jcmm.14606

18. Michels M, Ávila P, Pescador B, et al (2019) Microglial Cells Depletion Increases Inflammation and Modifies Microglial Phenotypes in an Animal Model of Severe Sepsis. Mol Neurobiol 56:7296–7304. https://doi.org/10.1007/s12035-019-1606-2

19. Milioli MVM, Burger H, Olivieri R, et al (2019) The impact of age on long-term behavioral and neurochemical parameters in an animal model of severe sepsis. Neurosci Lett 708:134339. https://doi.org/10.1016/j.neulet.2019.134339

20. Petronilho F, Tenfen L, Della Giustina A, et al (2019) Gold nanoparticles potentiates N-acetylcysteine effects on neurochemicals alterations in rats after polymicrobial sepsis. J Drug Target 28:428–436. https://doi.org/10.1080/1061186X.2019.1678168

21. Wu J, Liu H, Chu T, et al (2019) Neuregulin-1β attenuates sepsis-induced diaphragm atrophy by activating the PI3K/Akt signaling pathway. J Muscle Res Cell Motil 40:43–51. https://doi.org/10.1007/s10974-019-09512-2

22. Yang Y-M, Li Y-H, Ding L-L, et al (2019) Regulatory effect of lncRNA NKILA on autophagy induced by sepsis kidney injury. Eur Rev Med Pharmacol Sci 23:8011–8017. https://doi.org/10.26355/eurrev_201909_19017

23. Yang Y, Liang S, Li Y, et al (2019) Effects of early administration of insulin-like growth factor-1 on cognitive function in septic encephalopathy. Neuropsychiatr Dis Treat 15:323–337. https://doi.org/10.2147/NDT.S190845
