# Supplementary material for: Analysis of blood culture in a rat model of cecal ligation and puncture induced sepsis
Source: Intensive Care Med Exp. 2020 Jun 5;8:18. doi: 10.1186/s40635-020-00310-6 (PMC7275103; doi:10.1186/s40635-020-00310-6)
Supplement: Supplementary file 3 — Additional file 3: Table S2. Probabilistic antimicrobial therapy used in mouse CLP models in the literature published in 2018 and 2019 [file 40635_2020_310_MOESM3_ESM.docx]

Suppl Table2. Probabilistic antimicrobial therapy used in mouse CLP models in the

literature published in 2018 and 2019

| Author | Treatment, dose | Route of administration | | Duration | Objective |
| --- | --- | --- | --- | --- | --- |
| Al Zoubi S^1^  2018 | imipenem/cilastatin, 20 mg/kg | | SC | 6 and 18 h after CLP | Hypothesize that type 2 diabetes (T2DM) augments the cardiac (organ) dysfunction associated with sepsis, and that inhibition of the NF-κB pathway with linagliptin attenuates the cardiac (organ) dysfunction in mice with T2DM/sepsis. |
| Arimoto A^2^  2018 | Meropenem  (dose not specified) | | IP | Every 12 h for 3 days | Hypothesize that peritonitis induces immunosuppression in the lung, which in turn promotes lung metastasis |
| Ayalon I^3^  2018 | Imipenem 25 mg/kg | | IP | Single dose | Hypothesize that sepsis induces browning of the WAT (white adipose tissue) but obesity alters this adipose tissue response during sepsis. |
| Busch D^4^  2018 | Imipenem 25 mg/kg | | SC | Every 12 h until d 5 | Hypothesize that the PPAR-β/δ-agonist GW0742 improves long-term outcome after sepsis |
| Castanheira FVES^5^  2018 | Ertapenem 20 mg/kg | | IP | Beginning 6 h after CLP and then every 12 h up to day 3. | Hypothesize that ACKR2 could contribute to the degradation of chemokines in the foci of infection or in the vital organs, leading, in the first case, to aggravation of sepsis |
| Chen G^6^  2018 | combination of vancomycin (1 g/L), kanamycin (1 g/L), ampicillin (1 g/L), and metronidazole (1 g/L) in their drinking water | | PO | 2 months | Investigate the function of GPR109A in more detail, our study used cecum ligation and puncture surgery (CLP), described as the “gold standard” model for sepsis(26), to examine the effects of Gpr109a deletion. |
| de Souza Gomes R^7^  2018 | ceftriaxone 20 mg/kg | | IP | 24 h before and at time zero of CLP induction | Investigate the effect of pretreatment with salivary gland extract (SGE) from Ae. aegypti in the induction of inflammatory and oxidative processes |
| Drechsler S^8^  2018 | Imipenem 25 mg/kg | | SC | Twice daily for five consecutive days post-CLP | Investigate the effects of splenectomy as an added traumatic insult in a 2-hit mouse model of polytrauma (hemorrhagic shock with femur fracture) followed by abdominal sepsis after 48 hours |
| Fay KT^9^  2018 | ceftriaxone 25mg/kg + metronidazole 12.5mg/kg | | SC | 12h dosing schedule for 48 hours postoperatively. | utilize a CD43-/- model to further understand the impact of CD43 and the immunological derangements that occur in the senescent phase of sepsis. |
| Ferreira RG^10^  2018 | Ertapenem 20 mg/kg | | IP | Beginning 6 h after CLP and then every 12 h up to day 3. | Investigate the role of Gal-3 in sepsis outcome. |
| Gao R^11^  2018 | Ertapenem 20 mg/kg | | IP | Once daily for a total of 3 days | β2‑microglobulin in the brain also mediates sepsis‑induced cognitive impairment. |
| Guo L^12^  2018 | ceftriaxone 50 mg/kg + Meronidal 50 mg/kg | | IP | Every 12 h for 2 days | Explore the protective effects and understand the underlying mechanisms for the beneficial effects of live combined Bacillus subtilis and Enterococcus faecium (LCBE) in cecal ligation puncture (CLP)-induced sepsis. |
| Hu YM^13^  2018 | Ertapenem  75 mg/kg | | IP | Single dose 6 hours after surgery | Investigate glutamineeffects on regulating leukocyte programmed cell death 1 (PD-1) and its ligand (programmed cell death ligand 1 [PD-L1]) expression, macrophage function, inflammation, and acute kidney injury in sepsis. |
| Hu ZQ^14^  2018 | Imipenem-cilastatin 25 mg/kg | | SC | 6 hours after surgery and continuing every 12 h for 2 days | In the present study, a two-hit model which mimics clinical conditions was used and the potential role of Tregs in secondary Pseudomonas aeruginosa infection post-sepsis was investigated |
| Ji M^15^  2018 | Ertapenem 20 mg/kg | | SC | Immediately after operation | Hypothesize that p75NTR signaling might contribute to synaptic and cognitive impairments associated with sepsis induced by cecal ligation and perforation (CLP). |
| Ji MH^16^  2018 | Ertapenem 20 mg/kg | | SC | Single dose mmediately after operation | Investigate the impact of Mycophenolate mofetil on programmed death-1 expression within immune cells and its role in sepsis. |
| Jorge LB^17^  2018 | Imipenem-cilastatin 25 mg/kg | | SC | 6 hours and 18 hours after surgery | Analyze the role of Klotho in sepsis-induced AKI and multiple organ dysfunction |
| Karpurapu M^18^  2018 | Imipenem monohydrate supplemented water | | PO | During 96h | Identifiy a novel role for Nuclear Factors of Activated T cells (NFATc3) in the regulation of inflammatory genes produced by macrophages during murine sepsis-induced ALI |
| MacMillan-Crow LA^19^  2018 | Imipenem/cilastatin, 14 mg/kg | | SC | 6 hours and 18 hours after surgery | Investigate possible gender differences in renal mitochondrial function and dynamic |
| Matsuo S^20^  2018 | Imipenem/cilastatin  0,5 mg/kg; | | SC | Single dose after CLP | Hypothesize that administration of PYR-41, an inhibitor of ubiquitination, could reduce inflammation and organ injury in septic mice |
| Medeiros-de-Moraes IM^21^  2018 | imipenem 10 mg/kg | | IP | Single dose 6 hours after surgery | Analyze the effect of omega-9 supplementation on corticosteroid unbalance, inflammation, bacterial elimination, and peroxisome proliferator-activated receptor (PPAR) gamma expression, an omega-9 receptor and inflammatory modulator |
| Neves FS^22^  2018 | Ceftriaxone 30 mg/kg + clindamycin 25 mg/kg | | SC | Every 12 h for 3 days | Hypothesize that inhibition of brain insulin signaling might be associated with cognitive impairment observed in sepsis survivors |
| Ode Y^23^  2018 | Imipenem 0,5 µg/kg | | SC | Single dose | Hypothesize that CIRP induces ICAM-1 expression in neutrophils causing injury to the lungs during sepsis |
| Panpetch W^24^  2018 | imipenem/cilastatin, 14 mg/kg | | SC | Single dose 6 hours after surgery | Investigate that intestinal colonization with C albicans enhances bacterial sepsis severity through the gut translocation of BG in the absence of candidemia and that intestinal fungi suppression, by fluconazole, attenuates serum BG, mortality, and severity of inflammation as judged by serum IL-6. |
| Patel A^25^  2018 | Azithromycin 100 mg/kg i.p. +  ceftriaxone 100 mg/kg | | IP  SC | Single dose administered 3 h after CLP | Evaluate azithromycin benefit in the murine cecal ligation and puncture (CLP) model of polymicrobial sepsis. |
| Rana M^26^  2018 | imipenem/cilastatin, 0.5 mg/kg | | SC | Single dose after CLP | hypothesize that altered vagus nerve activity contributes to immune impairment in sepsis survivors |
| Steeland S^27^  2018 | Ceftriaxone 25 mg/kg + metronidazole 12.5 mg/kg | | SC | 9 and 24 h after CLP | Investigate plasma levels of both matrix metalloprotein-ase 8 and tumor necrosis factor receptor 1 are associated with sepsis severity and also investigates the therapeutic applicability of simultaneous inhibition of the two molecules in sepsis. |
| Taratummarat S^28^  2018 | Imipenem/cilastatin 14 mg/kg | | SC | Post-operation and at 6 h later. | Test if the administration of Gold nanoparticle, as an adjuvant to antibiotics, could attenuate bacterial sepsis |
| Xie J^29^  2018 | Ceftriaxone 25mg/kg + metronidazole 12.5mg/kg | | SC | Single dose after CLP | Hypothesize that the presence of pre-existing malignancy would result in phenotypic and functional changes in CD4+ T cell responses following sepsis |
| Xie Z^30^  2018 | Amoxicillin 10mg/kg | | IP | Single dose after CLP | Demonstrated the role of Wfdc21 in sepsis progression using a CLP-induced animal model and a cell-based mode |
| Xu H^31^  2018 | Imipenem-cilastatin 25 mg/kg | | SC | Every 12 h starting 2 h after CLP during 7 days | Test the hypothesis that IL‐33 is a regulator of lung inflammation in the early phases of sepsis. Using a model of polymicrobial intra‐abdominal sepsis, we showed that IL‐33 plays a central role in inducing lung injury within hours after the onset of severe sepsis. |
| Zhang H^32^  2018 | Imipenem-cilastatin 25 mg/kg | | SC | 8 and 16 h after CLP | Evaluate the immunological effect of miR-23b on NF-κB activity and the apoptotic signaling pathway. |
| Zhang H^33^  2018 | Imipenem-cilastatin 25 mg/kg | | SC | 8 and 16 h after CLP | Hypothesize that the induction of miR-23b in polymicrobial sepsis might emerge as a modifier to regulate fibrotic remodeling in the heart. |
| Zhou M^34^  2018 | Imipenem/cilastatin, 0.5 mg/kg | | SC | Single dose after CLP | Investigate the role of ghrelin for promoting the proliferation of CD4 T cells after sepsis |
| Zhou Y^35^  2018 | Imipenem 25 mg/kg | | SC | At 6, 24, and 48 h after CLP | Hypothesize that EPCs protect the microvasculature through the exosomes-mediated transfer of microRNAs (miRNAs). |
| Ballegeer M ^36^  2019 | Ceftriaxone 25 mg/kg and metronidazole 12.5 mg/kg. | | IP | 10 and 24 h after CLP onset | investigated the role of GILZ in sepsis |
| Barter J^37^  2019 | Imipenem 25 mg/kg | | SC | Starting 8 h after CLP and twice a day thereafter for a total of 2 doses in the mice sacrificed on postoperative day (POD) 1 and 6 doses  in mice sacrificed on  POD4 | determine how advanced age and sex influence the hippocampal transcriptome |
| Chen W^38^  2019 | Imipenem/cilastatin 0.5 mg/mouse | | SC | Single 30 min post CLP | Evaluate the effect of analgesics on sepsis-induced systemic inflammation |
| Das P^39^  2019 | Imipenem   1. 5 mg/kg 2. 25 mg/kg | | SC | a) 30 min after procedure (1 dose) to 10–12-week-  old mice  b) to 16–18-month-old mice every 12 h for 7 days | Investigate if Novel Chitohexaose Analog Protects Young and Aged mice from CLP Induced Polymicrobial Sepsis |
| Ding X^40^  2019 | Imipenem-cilastatine 25 mg/kg | | SC | Single dose after surgery | Hypothesize that Mechanical ventilation could exacerbate CLP-mediated lung injury through IL-33 and speculate that ventilation mode would correlate with IL-33 upregulation |
| Gong W^41^  2019 | Ertapenem 75 mg/kg beginning at | | IP | 6 h after surgery and reinjected every 24 h until day 3 post-surgery. | Use effective experimental models that faithfully replicate what occurs in patients with sepsis |
| Hoffman M^42^  2019 | Ertapenem 70 mg/kg | | IP | 2 hours after CLP and  every 24 hours durin 5  days | Investigate the course of cardiac dysfunction and myocardial load in different mouse models of sepsis to identify the optimal measurements for early systolic and diastolic dysfunction. |
| Hu Q^43^  2019 | Imipenem-cilastatin 25 mg/kg | | SC | 3 hours post-CLP | Hypothesise that STING is involved in the pathogenesis of sepsis by mediating IEC apoptosis induced by increased intestinal inflammation |
| Jones Buie JN^44^  2019 | Imipenem 25 mg/kg, | | SC | 6, 24, and 48 hours after CLP. | Hypothesize that cationic DEAC-pGlcNAc would form nanoparticles with miRNAs and thereby would successfully deliver active miRNA-126 with minimal toxicity and improve sepsis survival |
| Kitzmiller L^45^  2019 | Imipenem group 25 mg/kg | | IP | 1 h and every 24 h after the CLP procedure up to 72 h. | Investigate the effect of pharmacological activation of AMPK with A769662 on lung injury by using a model that would preferably mimic the clinical condition of adult patients |
| Kokkinaki D^46^  2019 | Ertapenem 70 mg/kg | | SC | 6 h after CLP and subsequently every 24 h for three days | Evaluated the therapeutic potential of the mammalian lignan precursor secoisolariciresinol diglucoside |
| Laroye C^47^  2019 | Imipenem 50 μg/g | | SC | Every 12 h for 7 days | hypothesize that MSC properties may vary depending on their tissue source in the setting of sepsis |
| Lyons JD^48^  2019 | Ceftriaxone 50 mg/kg + metronidazole 35 mg/kg | | SC | 12, 24, and 36 hours after surgery. | Determine the relative importance of pre-existing comorbidity and type of sepsis on the physiologic abnormalities induced by the combination of cancer and sepsis. |
| Mai C^49^  2019 | Clindamycin (150 mg/kg + ceftriaxone 50 mg/kg | | IP | Every 6 hours during 96h | Investigate the role of long non-coding RNA Lethe in mediating autophagy of cortical neurons in mice with sepsis-induced brain injury |
| Mei S^50^  2019 | Imipenem and cilastatin; | | SC | 14 mg/kg at 6 h and 7 mg/kg at 18 h after surgery | Prove whether mechanical ventilation with conventional tidal volume would aggravate multi-organ injuries induced by sepsis |
| Mella JR^51^  2019 | Imipenem 25 mg/kg | | SC | Beginning 2 hours after CLP and continued every 12 hours for a total of 6 doses | Hypothesize that NK-1R activation would contribute to the morbidity and mortality of sepsis in a model using mice genetically deficient in the NK-1R. |
| Naito Y^52^  2019 | Imipenem/cilastatin | | IP | 14 mg/kg 6 h after CLP AND repeated with 7 mg/kg every 12 h for 4 d | Identify candidate downstream factors of the TLR9 pathway that lead to septic acute kidney injury |
| O'Riordan^53^  2019 | Imipenem/cilastatin 20 mg/kg in saline | | SC | 6 and 18 h after surgery | Investigate whether pharmacological inhibition of BTK (ibrutinib 30 mg/kg and acalabrutinib 3 mg/kg) attenuates sepsis associated cardiac dysfunction in mice |
| Pang D^54^  2019 | Enrofloxacin 5 mg/kg | | SC | Twice daily during 4 days of the study | Examine the time course and location of early microglial activation and its anatomic relationship to the development of acute cerebral edema |
| Pereira PAT^55^  2019 | ErtapenemInc., 75 mg/kg | | IP | Beginning at 3 h after surgery and re-injected every 24 h until day 3 after surgery | Investigate survival outcomes, and immunological and metabolomic efects of hyaluronidase |
| Seymour CW^56^  2019 | Imipenem/cilastatin 25 mg/kg | | IP | Single dose i immediatly or 2h or 4h after physiological détérioration | Determine phenotypes of polymicrobial sepsis prior to physiologic deterioration, and the association between phenotypes and outcome in a randomized trial of prompt or delayed antibiotics and fluids. |
| Silva JF^57^  2019 | Ertapenem 30 mg/kg | | SC | 1 h after CLP and every 12 h, for 3 days | Investigate whether O-GlcNAc affects the inflammatory response and cardiovascular dysfunction associated with sepsis |
| Skirecki T^58^  2019 | Imipenem 25 mg/kg, | | SC | From 2 h post-CLP Twice daily (approximately every 12 h) for 5 consecutive days post-CLP. | Evaluate the early outcome-dependent immuno-inflammatory response in humanized mice |
| Umakoshi K^59^  2019 | Meropenem 100 mg/kg | | SC | 3 h after CLP and the subsequent every 12 h durin 7 days | investigate kinetic changes in the immune responses |
| Vandewalle J^60^  2019 | Ceftriaxone (25 mg/kg + metronidazole 12.5 mg/kg | | IP | Ten and 24 h after CLP | Hypothesize that TNF has a dual role in sepsis, namely a mediating and a protective role, and that protection might be obtained by TNFR1-specific inhibition. |
| Vu CTB^61^  2019 | imipénem-cilastatine 10mg/kg | | IV | Immediately after the operation, and at 6 h and 24 h later. | Determine kinetic changes in the immune phenotype by determining the proportion of T cells, B cells and macrophages, and especially the expression of an immune exhaustion marker PD |
| Zhang Z^62^  2019 | ceftriaxone 30 mg/kg + clindamycin 25 mg/kg | | SC | Every 12 h for a total of 3 days | Probenecid administration CAN inhibit PANX1-mediated release of cellular ATP and ameliorate cognitive impairment |

IP: intraperitoneal; IV: intravenous SC: subcutaneous; PO: per os

[1–62]

1. Al Zoubi S, Chen J, Murphy C, et al (2018) Linagliptin Attenuates the Cardiac Dysfunction Associated With Experimental Sepsis in Mice With Pre-existing Type 2 Diabetes by Inhibiting NF-κB. Front Immunol 9:2996. https://doi.org/10.3389/fimmu.2018.02996

2. Arimoto A, Yamashita K, Hasegawa H, et al (2018) Immunosuppression Induced by Perioperative Peritonitis Promotes Lung Metastasis. Anticancer Res 38:4333–4338. https://doi.org/10.21873/anticanres.12733

3. Ayalon I, Shen H, Williamson L, et al (2018) Sepsis Induces Adipose Tissue Browning in Nonobese Mice But Not in Obese Mice. Shock 50:557–564. https://doi.org/10.1097/SHK.0000000000001076

4. Busch D, Kapoor A, Rademann P, et al (2018) Delayed activation of PPAR-β/δ improves long-term survival in mouse sepsis: effects on organ inflammation and coagulation. Innate Immun 24:262–273. https://doi.org/10.1177/1753425918771748

5. Castanheira FVES, Borges V, Sônego F, et al (2018) The Atypical Chemokine Receptor ACKR2 is Protective Against Sepsis. Shock 49:682–689. https://doi.org/10.1097/SHK.0000000000000969

6. Chen G, Huang B, Fu S, et al (2018) G Protein-Coupled Receptor 109A and Host Microbiota Modulate Intestinal Epithelial Integrity During Sepsis. Front Immunol 9:2079. https://doi.org/10.3389/fimmu.2018.02079

7. de Souza Gomes R, Navegantes-Lima KC, Monteiro VVS, et al (2018) Salivary Gland Extract from Aedes aegypti Improves Survival in Murine Polymicrobial Sepsis through Oxidative Mechanisms. Cells 7:. https://doi.org/10.3390/cells7110182

8. Drechsler S, Zipperle J, Rademann P, et al (2018) Splenectomy modulates early immuno-inflammatory responses to trauma-hemorrhage and protects mice against secondary sepsis. Sci Rep 8:14890. https://doi.org/10.1038/s41598-018-33232-1

9. Fay KT, Chihade DB, Chen C-W, et al (2018) Increased mortality in CD43-deficient mice during sepsis. PLoS ONE 13:e0202656. https://doi.org/10.1371/journal.pone.0202656

10. Ferreira RG, Rodrigues LC, Nascimento DC, et al (2018) Galectin-3 aggravates experimental polymicrobial sepsis by impairing neutrophil recruitment to the infectious focus. J Infect 77:391–397. https://doi.org/10.1016/j.jinf.2018.06.010

11. Gao R, Li G, Yang R, et al (2018) Hippocampal β2‑microglobulin mediates sepsis‑induced cognitive impairment. Mol Med Rep 17:7813–7820. https://doi.org/10.3892/mmr.2018.8858

12. Guo L, Meng M, Wei Y, et al (2018) Protective Effects of Live Combined B. subtilis and E. faecium in Polymicrobial Sepsis Through Modulating Activation and Transformation of Macrophages and Mast Cells. Front Pharmacol 9:1506. https://doi.org/10.3389/fphar.2018.01506

13. Hu Y-M, Hsiung Y-C, Pai M-H, Yeh S-L (2018) Glutamine Administration in Early or Late Septic Phase Downregulates Lymphocyte PD-1/PD-L1 Expression and the Inflammatory Response in Mice With Polymicrobial Sepsis. JPEN J Parenter Enteral Nutr 42:538–549. https://doi.org/10.1177/0148607117695245

14. Hu Z-Q, Yao Y-M, Chen W, et al (2018) Partial Depletion of Regulatory T Cells Enhances Host Inflammatory Response Against Acute Pseudomonas aeruginosa Infection After Sepsis. Inflammation 41:1780–1790. https://doi.org/10.1007/s10753-018-0821-8

15. Ji M, Yuan H, Yuan S, et al (2018) The p75 neurotrophin receptor might mediate sepsis-induced synaptic and cognitive impairments. Behav Brain Res 347:339–349. https://doi.org/10.1016/j.bbr.2018.03.042

16. Ji M-H, Xia D-G, Zhu L-Y, et al (2018) Short- and Long-Term Protective Effects of Melatonin in a Mouse Model of Sepsis-Associated Encephalopathy. Inflammation 41:515–529. https://doi.org/10.1007/s10753-017-0708-0

17. Jorge LB, Coelho FO, Sanches TR, et al (2019) Klotho deficiency aggravates sepsis-related multiple organ dysfunction. Am J Physiol Renal Physiol 316:F438–F448. https://doi.org/10.1152/ajprenal.00625.2017

18. Karpurapu M, Lee YG, Qian Z, et al (2018) Inhibition of nuclear factor of activated T cells (NFAT) c3 activation attenuates acute lung injury and pulmonary edema in murine models of sepsis. Oncotarget 9:10606–10620. https://doi.org/10.18632/oncotarget.24320

19. MacMillan-Crow LA, Mayeux PR (2018) Female mice exhibit less renal mitochondrial injury but greater mortality using a comorbid model of experimental sepsis. Intern Med Rev (Wash D C) 4:. https://doi.org/10.18103/imr.v4i10.768

20. Matsuo S, Sharma A, Wang P, Yang W-L (2018) PYR-41, A Ubiquitin-Activating Enzyme E1 Inhibitor, Attenuates Lung Injury in Sepsis. Shock 49:442–450. https://doi.org/10.1097/SHK.0000000000000931

21. Medeiros-de-Moraes IM, Gonçalves-de-Albuquerque CF, Kurz ARM, et al (2018) Omega-9 Oleic Acid, the Main Compound of Olive Oil, Mitigates Inflammation during Experimental Sepsis. Oxid Med Cell Longev 2018:6053492. https://doi.org/10.1155/2018/6053492

22. Neves FS, Marques PT, Barros-Aragão F, et al (2018) Brain-Defective Insulin Signaling Is Associated to Late Cognitive Impairment in Post-Septic Mice. Mol Neurobiol 55:435–444. https://doi.org/10.1007/s12035-016-0307-3

23. Ode Y, Aziz M, Wang P (2018) CIRP increases ICAM-1+ phenotype of neutrophils exhibiting elevated iNOS and NETs in sepsis. J Leukoc Biol 103:693–707. https://doi.org/10.1002/JLB.3A0817-327RR

24. Panpetch W, Somboonna N, Bulan DE, et al (2018) Gastrointestinal Colonization of Candida Albicans Increases Serum (1→3)-β-D-Glucan, without Candidemia, and Worsens Cecal Ligation and Puncture Sepsis in Murine Model. Shock 49:62–70. https://doi.org/10.1097/SHK.0000000000000896

25. Patel A, Joseph J, Periasamy H, Mokale S (2018) Azithromycin in Combination with Ceftriaxone Reduces Systemic Inflammation and Provides Survival Benefit in a Murine Model of Polymicrobial Sepsis. Antimicrob Agents Chemother 62:. https://doi.org/10.1128/AAC.00752-18

26. Rana M, Fei-Bloom Y, Son M, et al (2018) Constitutive Vagus Nerve Activation Modulates Immune Suppression in Sepsis Survivors. Front Immunol 9:2032. https://doi.org/10.3389/fimmu.2018.02032

27. Steeland S, Van Ryckeghem S, Vandewalle J, et al (2018) Simultaneous Inhibition of Tumor Necrosis Factor Receptor 1 and Matrix Metalloproteinase 8 Completely Protects Against Acute Inflammation and Sepsis. Crit Care Med 46:e67–e75. https://doi.org/10.1097/CCM.0000000000002813

28. Taratummarat S, Sangphech N, Vu CTB, et al (2018) Gold nanoparticles attenuates bacterial sepsis in cecal ligation and puncture mouse model through the induction of M2 macrophage polarization. BMC Microbiol 18:85. https://doi.org/10.1186/s12866-018-1227-3

29. Xie J, Robertson JM, Chen C-W, et al (2018) Pre-existing malignancy results in increased prevalence of distinct populations of CD4+ T cells during sepsis. PLoS ONE 13:e0191065. https://doi.org/10.1371/journal.pone.0191065

30. Xie Z, Guo Z, Liu J (2018) Whey Acidic Protein/Four-Disulfide Core Domain 21 Regulate Sepsis Pathogenesis in a Mouse Model and a Macrophage Cell Line via the Stat3/Toll-Like Receptor 4 (TLR4) Signaling Pathway. Med Sci Monit 24:4054–4063. https://doi.org/10.12659/MSM.907176

31. Xu H, Xu J, Xu L, et al (2018) Interleukin-33 contributes to ILC2 activation and early inflammation-associated lung injury during abdominal sepsis. Immunol Cell Biol 96:935–947. https://doi.org/10.1111/imcb.12159

32. Zhang H, Caudle Y, Shaikh A, et al (2018) Inhibition of microRNA-23b prevents polymicrobial sepsis-induced cardiac dysfunction by modulating TGIF1 and PTEN. Biomed Pharmacother 103:869–878. https://doi.org/10.1016/j.biopha.2018.04.092

33. Zhang H, Li H, Shaikh A, et al (2018) Inhibition of MicroRNA-23b Attenuates Immunosuppression During Late Sepsis Through NIK, TRAF1, and XIAP. J Infect Dis 218:300–311. https://doi.org/10.1093/infdis/jiy116

34. Zhou M, Aziz M, Ochani M, et al (2018) The protective role of human ghrelin in sepsis: Restoration of CD4 T cell proliferation. PLoS ONE 13:e0201139. https://doi.org/10.1371/journal.pone.0201139

35. Zhou Y, Li P, Goodwin AJ, et al (2018) Exosomes from Endothelial Progenitor Cells Improve the Outcome of a Murine Model of Sepsis. Mol Ther 26:1375–1384. https://doi.org/10.1016/j.ymthe.2018.02.020

36. Ballegeer M, Vandewalle J, Eggermont M, et al (2019) Overexpression of Gilz Protects Mice Against Lethal Septic Peritonitis. Shock 52:208–214. https://doi.org/10.1097/SHK.0000000000001252

37. Barter J, Kumar A, Stortz JA, et al (2019) Age and Sex Influence the Hippocampal Response and Recovery Following Sepsis. Mol Neurobiol 56:8557–8572. https://doi.org/10.1007/s12035-019-01681-y

38. Chen W, Brenner M, Aziz M, et al (2019) Buprenorphine Markedly Elevates a Panel of Surrogate Markers in a Murine Model of Sepsis. Shock 52:550–553. https://doi.org/10.1097/SHK.0000000000001361

39. Das P, Panda SK, Agarwal B, et al (2019) Novel Chitohexaose Analog Protects Young and Aged mice from CLP Induced Polymicrobial Sepsis. Sci Rep 9:2904. https://doi.org/10.1038/s41598-019-38731-3

40. Ding X, Jin S, Shao Z, et al (2019) The IL-33-ST2 Pathway Contributes to Ventilator-Induced Lung Injury in Septic Mice in a Tidal Volume-Dependent Manner. Shock 52:e1–e11. https://doi.org/10.1097/SHK.0000000000001260

41. Gong W, Wen H (2019) Sepsis Induced by Cecal Ligation and Puncture. Methods Mol Biol 1960:249–255. https://doi.org/10.1007/978-1-4939-9167-9_22

42. Hoffman M, Kyriazis ID, Lucchese AM, et al (2019) Myocardial Strain and Cardiac Output are Preferable Measurements for Cardiac Dysfunction and Can Predict Mortality in Septic Mice. J Am Heart Assoc 8:e012260. https://doi.org/10.1161/JAHA.119.012260

43. Hu Q, Ren H, Li G, et al (2019) STING-mediated intestinal barrier dysfunction contributes to lethal sepsis. EBioMedicine 41:497–508. https://doi.org/10.1016/j.ebiom.2019.02.055

44. Jones Buie JN, Zhou Y, Goodwin AJ, et al (2019) Application of Deacetylated Poly-N-Acetyl Glucosamine Nanoparticles for the Delivery of miR-126 for the Treatment of Cecal Ligation and Puncture-Induced Sepsis. Inflammation 42:170–184. https://doi.org/10.1007/s10753-018-0882-8

45. Kitzmiller L, Ledford JR, Hake PW, et al (2019) Activation of AMP-Activated Protein Kinase by A769662 Ameliorates Sepsis-Induced Acute Lung Injury in Adult Mice. Shock 52:540–549. https://doi.org/10.1097/SHK.0000000000001303

46. Kokkinaki D, Hoffman M, Kalliora C, et al (2019) Chemically synthesized Secoisolariciresinol diglucoside (LGM2605) improves mitochondrial function in cardiac myocytes and alleviates septic cardiomyopathy. J Mol Cell Cardiol 127:232–245. https://doi.org/10.1016/j.yjmcc.2018.12.016

47. Laroye C, Boufenzer A, Jolly L, et al (2019) Bone marrow vs Wharton’s jelly mesenchymal stem cells in experimental sepsis: a comparative study. Stem Cell Res Ther 10:192. https://doi.org/10.1186/s13287-019-1295-9

48. Lyons JD, Chen C-W, Liang Z, et al (2019) Murine Pancreatic Cancer Alters T Cell Activation and Apoptosis and Worsens Survival After Cecal Ligation and Puncture. Shock 51:731–739. https://doi.org/10.1097/SHK.0000000000001203

49. Mai C, Qiu L, Zeng Y, Jian H-G (2019) LncRNA Lethe protects sepsis-induced brain injury via regulating autophagy of cortical neurons. Eur Rev Med Pharmacol Sci 23:4858–4864. https://doi.org/10.26355/eurrev_201906_18073

50. Mei S, Wang S, Jin S, et al (2019) Human Adipose Tissue-Derived Stromal Cells Attenuate the Multiple Organ Injuries Induced by Sepsis and Mechanical Ventilation in Mice. Inflammation 42:485–495. https://doi.org/10.1007/s10753-018-0905-5

51. Mella JR, Stucchi AF, Duffy ER, Remick DG (2019) Neurokinin-1 Receptor Deficiency Improves Survival in Murine Polymicrobial Sepsis Through Multiple Mechanisms in Aged Mice. Shock 52:61–66. https://doi.org/10.1097/SHK.0000000000001248

52. Naito Y, Tsuji T, Nagata S, et al (2020) IL-17A activated by Toll-like receptor 9 contributes to the development of septic acute kidney injury. Am J Physiol Renal Physiol 318:F238–F247. https://doi.org/10.1152/ajprenal.00313.2019

53. O’Riordan CE, Purvis GSD, Collotta D, et al (2019) Bruton’s Tyrosine Kinase Inhibition Attenuates the Cardiac Dysfunction Caused by Cecal Ligation and Puncture in Mice. Front Immunol 10:2129. https://doi.org/10.3389/fimmu.2019.02129

54. Pang D, Wu YL, Alcamo AM, et al (2019) Early Axonal Injury and Delayed Cytotoxic Cerebral Edema are Associated With Microglial Activation in a Mouse Model of Sepsis. Shock. https://doi.org/10.1097/SHK.0000000000001446

55. Pereira PAT, Bitencourt CS, Reis MB, et al (2020) Immunomodulatory activity of hyaluronidase is associated with metabolic adaptations during acute inflammation. Inflamm Res 69:105–113. https://doi.org/10.1007/s00011-019-01297-x

56. Seymour CW, Kerti SJ, Lewis AJ, et al (2019) Murine sepsis phenotypes and differential treatment effects in a randomized trial of prompt antibiotics and fluids. Crit Care 23:384. https://doi.org/10.1186/s13054-019-2655-7

57. Silva JF, Olivon VC, Mestriner FLAC, et al (2019) Acute Increase in O-GlcNAc Improves Survival in Mice With LPS-Induced Systemic Inflammatory Response Syndrome. Front Physiol 10:1614. https://doi.org/10.3389/fphys.2019.01614

58. Skirecki T, Drechsler S, Hoser G, et al (2019) The Fluctuations of Leukocytes and Circulating Cytokines in Septic Humanized Mice Vary With Outcome. Front Immunol 10:1427. https://doi.org/10.3389/fimmu.2019.01427

59. Umakoshi K, Choudhury ME, Nishioka R, et al (2020) B lymphocytopenia and Bregs in a not-to-die murine sepsis model. Biochem Biophys Res Commun 523:202–207. https://doi.org/10.1016/j.bbrc.2019.12.041

60. Vandewalle J, Steeland S, Van Ryckeghem S, et al (2019) A Study of Cecal Ligation and Puncture-Induced Sepsis in Tissue-Specific Tumor Necrosis Factor Receptor 1-Deficient Mice. Front Immunol 10:2574. https://doi.org/10.3389/fimmu.2019.02574

61. Vu CTB, Thammahong A, Leelahavanichkul A, Ritprajak P (2019) Alteration of macrophage immune phenotype in a murine sepsis model is associated with susceptibility to secondary fungal infection. Asian Pac J Allergy Immunol. https://doi.org/10.12932/AP-170519-0565

62. Zhang Z, Lei Y, Yan C, et al (2019) Probenecid Relieves Cerebral Dysfunction of Sepsis by Inhibiting Pannexin 1-Dependent ATP Release. Inflammation 42:1082–1092. https://doi.org/10.1007/s10753-019-00969-4
